# Cornstalk Diseases1Read before the Nebraska Veterinary Medical Association, February 21, 1899.

**Published:** 1899-06

**Authors:** A. Bostrom

**Affiliations:** Minden, Nebraska


### CORNSTALK DISEASES.[1]

By A. Bostrom, D.V.S.,
MINDEN, NEBRASKA.

I will make an attempt to call your attention to a few facts concerning that inexhaustible subject, cornstalk disease.

The cornstalk is looked upon with suspicion as an agent which sometimes seems to be incompatible with animal life; yet thousands of horses and cattle are turned out to feed upon it every year, and if anything happens to interfere with the natural laws which govern health and life of the animals the cornstalk is held responsible for it; and the question is, What is the matter with the stalks?

Before trying to answer this question, let us consider the corn-plant in detail. I believe that the corn-plant is subject to the natural laws which govern the vegetable kingdom. We plant the seed, it germinates and grows; we cultivate it, and it gets ripe; we take care of the seeds, and the stalk is left in the field to take care of itself. It is subject to diseases like other members of the

[1] Read before the Nebraska Veterinary Medical Association, February 21, 1899.

vegetable kingdom. Both animal and vegetable parasites may affect its growth, of which the vegetable parasites are the most important group, including such pests as smut, caused by the fungus *Ustilago carbo* and the *Ustilago maydis;* rust, of which the most important is *Puccinia maydis;* and ergot, a fungoid disease not only affecting the rye-plant, but many other species of gramina, such as corn, wheat, etc.

About the time of maturity the most tender leaves of the plant begin to fall off upon the ground, and there they become covered with small fungi of the genus *Penicellium, Aspergillus, Mucor, Ascophora,* etc. Small sideshoots, developing only rudimentary ears, die early, and in going through the process of decay contain innumerable numbers of the above-named fungi. Now this is the actual condition of the corn-plant as near as I can describe it.

Burrill, Billings, and others say that they have discovered a germ that is the cause of the cornstalk disease. Others say that the cornstalk contains so much saltpetre when grown in certain places that the animals eating it die of saltpetre-poisoning.

Now, after considering the various conditions of the corn-plant, let us turn our attention to some of the other plants for comparison.

Clover is subject to the attacks of several fungi, of which the most important is the *Peronospora trifolium,* which exerts its irritating action directly upon the gastro-intestinal mucous membrane, or the formation of a toxic substance which, acting particularly upon the liver and the brain, causes what is known as clover-disease. Straw of wheat and oats, when damp and going through a mouldy change, is affected by various fungi, such as *Tilletia, Caries, Puccinia, Graminis,* etc., causing derangement of the intestinal and urinary organs, followed by paralysis and death.

We have already seen that the cornstalks are sometimes affected by various fungi, and I am inclined to believe that if the fungi on clover produce clover-disease the fungi on cornstalks are the cause of the so-called cornstalk disease. I have seen both horses and cattle die from eating straw covered with fungi. I have seen hogs die from the effect of mouldy flour. I have had considerable experience with the so-called cerebritis or encephalitis, described in our veterinary publications as being caused by wormy and mouldy corn, and I believe that all these conditions, the cornstalk disease included, are cases of fungus-poisoning.

Cornstalks may be the cause of other conditions which frequently result in death. Sometimes cornstalks are cut up and fed to cattle and horses in July and August, when grass in the pasture is insufficient, and when fed fresh and in limited amounts I have never seen or heard of any bad results therefrom; but when the stalks have been cut at this time of the year and allowed to remain in the field for a week or ten days—long enough to allow the development of an active process of fermentation—and then fed, I have seen the most serious disease, with death following in one-half to two hours of *acute tympanitis* or *meteorism*. If seen in time the animal can be relieved by tapping. After the escape of the gas the animal gets well in a very short time. I do not believe that any other germ except the bacteria of fermentation could be the cause of the production of this gas; and the fact that the tapping and the escape of the gas left the animal well in such a short time is proof enough that this is not a pathogenic bacterial disease.

Acute indigestion and gastritis, with or without engorgement, with metastasis to the brain through reflex nervous action, is a frequent occurrence in the stalk-fields, and I believe that neither the Burrill-Billings bacillus nor saltpetre have anything to do with it. Both horses and cattle are liable to overload themselves if allowed to have free access to the stalk-fields, especially when first turned into the field. Cases of engorgement of this kind are often followed by paralysis and death, especially in cows. Now, regarding saltpetre-poisoning, the question is, Is it possible that the corn-plant *can* absorb such abnormal amounts of saltpetre, even if the ground upon which it grows should happen to be very rich in saltpetre? The statistics of chemical analysis of the composition of various plants are regarded as facts by which we can determine the amount of the various kinds of food and in what proportion they should be supplied in order to get a certain result. If it is a fact that the cornstalk, or any other plant growing under natural conditions, is of such a nature that it is a good, wholesome plant when grown in certain places, but a poisonous one if grown in another, simply because that place happened to contain too much of one of the natural ingredients of that plant, then the figures of chemical analysis are very unreliable.

Now we have seen that the cornstalk may be the cause of *acute tympanitis*, *indigestion*, *gastritis*, and *fungus-poisoning*, simply because it is often eaten in excessive quantity or in bad quality. Straw, hay, grain, and flour fed in the same condition will produce the same result; but it is a fact that while we generally take care of and save all other plants used for animal food, the cornstalk is left in the field to take care of itself, under the influence of all kinds of winds and weather. The dry stalks which stand there in defiance to all the elements of nature have become a mass of woody fibres, good for nothing except to cause indigestion.

The most tender parts, such as leaves and sideshoots, fall upon the ground, there to go through the process of decay, the ultimate process of reduction to its original state—dust. These rotten parts are generally eaten by the young stock, going through the active process of dentition, in preference to the other parts which require active mastication; and the young animal, which has eaten almost nothing but these decaying, mouldy parts, dies of *fungus-poisoning;* the older stock more frequently die from *indigestion.* Early in the fall I think there is some nutriment in the stalks, and there is generally some corn left in the field; and I believe that if cattle and horses are allowed to eat a limited amount at a time they will do well on it, provided that the organs of digestion are in good order when this sudden change of diet takes place. Mild cases of fungus-poisoning we frequently notice as toxic polyuria, caused by mouldy feed.

As an accessory cause in the development of the diseases now spoken of I will particularly mention *cold.* Cold acts as a debilitant if long continued or severe. It weakens the circulation, especially that of the surface of the body, causes internal congestion, and directly lowers all the vital energies.

In conclusion, I will call your attention to the fact that of all the disease-producing causes there is no single factor which has so much influence as the quality of food, and I believe that the cornstalk disease, as well as many other diseases, is the result of the violation or non-application of the rules of hygiene. All plants intended for animal food should be cut when they are ripe, dried, and saved properly. Water should be pure, plenty of it, and accessible at all times. Salt at least twice a week. Good shelter and plenty of bedding. When this is observed disease will be reduced to a minimum.

